# Post-diapause synthesis of ArHsp40-2, a type 2 J-domain protein from *Artemia franciscana*, is developmentally regulated and induced by stress

**DOI:** 10.1371/journal.pone.0201477

**Published:** 2018-07-26

**Authors:** Nathan M. Rowarth, Thomas H. MacRae

**Affiliations:** Department of Biology, Dalhousie University, Halifax, NS, Canada; National Center for Toxicological Research, UNITED STATES

## Abstract

Post-diapause cysts of *Artemia franciscana* undergo a well-defined developmental process whereby internal differentiation leads to rupture of the cyst shell, release of membrane-enclosed nauplii and hatching to yield swimming larvae. The post-diapause development of *A*. *franciscana* has been examined at biochemical and molecular levels, yet little is known about molecular chaperone function during this process. In addressing this we recently described ArHsp40, a type 1 J-domain protein in post-diapause *A*. *franciscana* cysts and larvae. The current report describes ArHsp40-2, a second J-domain protein from *A*. *franciscana*. ArHsp40-2 is a type 2 J-domain protein, lacking a zinc binding domain but containing other domains characteristic of these proteins. Notably, ArHsp40-2 possesses a double barrel β-domain structure in its substrate binding region, as does ArHsp40. qPCR revealed a relatively low amount of *ArHsp40-2* mRNA in 0 h cysts which increased significantly until the E1 stage, most likely as a result of enhanced transcription, after which it declined. An antibody specific to ArHsp40-2 was produced and used to show that like its mRNA, ArHsp40-2 accumulated until the E1 stage and then decreased to amounts lower than those in 0 h cysts. The synthesis of ArHsp40-2 was induced by heat shock indicating that ArHsp40-2 is involved in stress resistance in cysts and nauplii. Accumulation in cysts during early post-diapause development followed by its sharp decline suggests a role in protein disaggregation/refolding, a function of Hsp40s from other organisms, where ArHsp40-2 assists in the rescue of proteins sequestered during diapause by p26, an abundant small heat shock protein (sHsp) in *A*. *franciscana* cysts.

## Introduction

Fertilized eggs of the extremophile crustacean, *Artemia franciscana* develop ovoviviparously in the female’s brood sac and at five days post-fertilization swimming nauplii (larvae) are released [1, 2]. Under optimal conditions larvae molt several times and reach sexual maturity in four to five weeks. With changing parameters such as photoperiod and temperature, oviparous development ensues, during which embryos arrest as gastrulae comprised of approximately 4000 cells and are coated with a chitinous shell impermeable to non-volatile compounds [3, 4]. The encysted embryos, termed cysts, are released from the brood sac and after six to eight days enter diapause, a physiological state of reduced metabolism and enhanced stress tolerance that allows survival under adverse environmental conditions [4–7]. Once diapause terminates the encysted embryos resume growth if conditions are favourable and the resulting nauplii develop in the same way as nauplii arising ovoviviparously.

Cysts of *A*. *franciscana* possess a small heat shock protein (sHsp) termed p26, an abundant ATP-independent molecular chaperone constituting 7% of soluble cyst protein [1, 8, 9]. As shown by RNA interference (RNAi), p26 provides stress protection [10], and as for other sHsps [11–16], p26 is thought to bind a wide range of aberrant proteins and prevent their irreversible denaturation. Although sHsps bind to and protect proteins during stress, such as that experienced during diapause, they are unable to refold these substrates. Removal of proteins from sHsps and their refolding, as well as the solubilisation of protein aggregates, requires ATP-dependent molecular chaperones including Hsp70/Hsc70, Hsp110, Hsp90 and Hsp40, which form multi-protein complexes [16–19]. Within these functional chaperone networks Hsp40s, also known as J-domain proteins, are major co-chaperones of Hsp70 and they facilitate the delivery of non-native proteins to Hsp70 and enhance its ATPase activity [20, 21]. Additionally, Hsp40s aid in disaggregation and the subsequent refolding of proteins, processes that involve Hsp70 and Hsp110, the latter a nucleotide exchange factor [16, 17, 19, 21–23]. *In vitro* studies using heat aggregated luciferase and *in vivo* studies with *Caenorhabditis elegans* show that the efficiency of the disaggregase/refolding system is greatest when type 1 and type 2 Hsp40s cooperate [19, 24].

The J-domain proteins constitute a diverse family generally divided into three main groups, types 1, 2 and 3 or A, B and C, although this classification scheme is not likely adequate to capture their structural complexity [21]. All J-domain proteins possess a J-domain containing a highly conserved HPD motif which activates Hsp70 ATPase [25, 26]. Type 1 J-domain proteins have an amino-terminal J-domain, followed by a G/F rich region that may recognize substrate proteins and facilitate Hsp70 binding, a zinc-binding domain for interaction with substrates that is characterized by four CXXCXGXG motifs and a carboxyl-terminal domain for dimer formation with other J-domain proteins. Type 2 J-domain proteins are similar to Type 1 but do not have a zinc-binding domain. Type 3 J-domain proteins, each limited in its range of clients, contain a J-domain of variable location, but lack a G/F rich region and a zinc-binding domain [27, 28].

ArHsp40, a type 1 J-domain protein from *A*. *franciscana* has been described [29] but no other J-domain proteins are reported for this organism. To approach the question of Hsp40 diversity and function in *A*. *franciscana*, and to investigate its relationship, if any, with p26 during post-diapause development, a search was made for additional J-domain proteins. As a consequence, a type 2 J-domain protein termed ArHsp40-2, which lacks a zinc binding domain, was found. The synthesis of ArHsp40-2, like ArHsp40 [29] was heat inducible, indicating a role in stress tolerance. However the synthesis pattern of ArHsp40-2 during post-diapause development was different than that of ArHsp40 and the time of maximum accumulation suggested a role for ArHsp40-2 in the recovery of proteins during post-diapause development.

## Materials and methods

### Culture of *A*. *franciscana*

**Fig 1:**
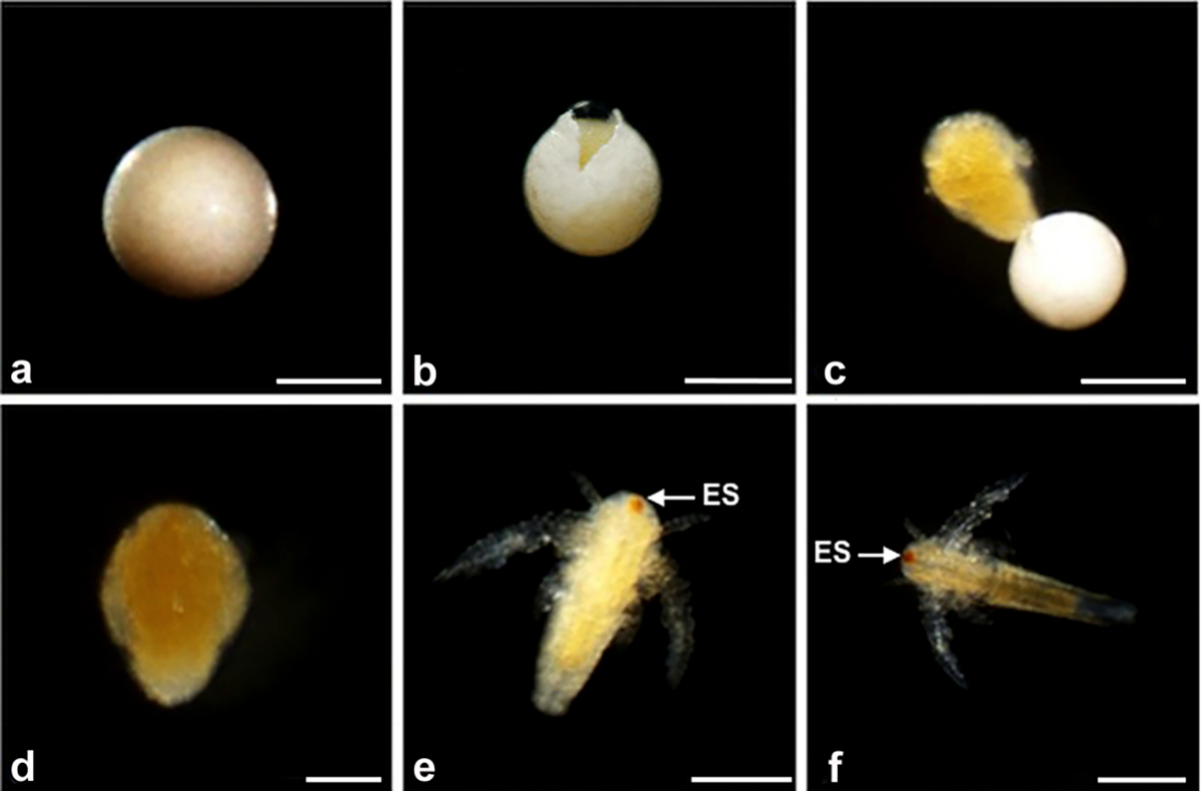
Post-diapause life history stages of *A*. *franciscana*. Light micrographs of *A*. *franciscana* obtained with a Nikon AZ100 microscope. (a) Cysts prior to emergence (0 h); (b) emerged nauplius 1 (E1); (c) emerged nauplius 2 (E2); (d) emerged nauplius 3 (E3); (e) instar 1 nauplius; (f) early instar 2 nauplius. Early and late (not shown) instar 2 nauplii are morphologically similar. ES, eye spot. Magnification bar a, 100 μm; b, c, 250 μm; d—f, 100 μm.

*A*. *franciscana* cysts from the Great Salt Lake (GSL) (INVE Aquaculture Inc., Ogden, UT, USA), hydrated for at least 3 h on ice in distilled H_2_O and collected by suction filtration, were incubated at room temperature in 1 μm filtered, UV treated and autoclaved 33.0 ppt saltwater from Halifax Harbor, NS, Canada, hereafter termed seawater. Animals were collected for mRNA and protein preparation following hydration (0 h cysts), after 5 h at 27°C (5 h cysts), after 10 h at 27 ^o^C (10 h cysts/E1 nauplii) and as completely emerged, membrane-enclosed E2/E3 nauplii (Fig 1). After the hatching of E2/E3 nauplii, 1^st^ instar larvae were collected by photo-taxis [29, 30] and either homogenized immediately or incubated at 27°C for either 16 h or 26 h to respectively generate early and late 2^nd^ instar larvae (Fig 1). The research described in this paper was performed in accordance with the ethical guidelines provided by the Canadian Council on Animal Care (CCAC). The University Committee on Laboratory Animals (UCLA) of Dalhousie University approved the research and assigned the Protocol Number 117–36.

### Cloning and sequencing of *A*. *franciscana* type 2 Hsp40 cDNA

One mg of cysts developed for 5 h was harvested from seawater on 5 μm nylon mesh filters (Spectrum Labs Inc., Rancho Dominguez, CA, USA), flash frozen in liquid nitrogen and then homogenized in 500 μl of TRIzol® (Invitrogen, Burlington, ON, Canada). RNA was extracted according to manufacturer’s instructions. Single-strand cDNA was synthesized with the SuperScript® III First-Strand Synthesis System for RT-PCR (Invitrogen, Burlington, ON, Canada). RNA was quantified by measuring the absorbance at 260 nm and 0.1 μg mRNA was used as template for synthesizing cDNA with SuperScript® III First-Strand Synthesis Kit for RT-PCR (Invitrogen, Burlington, ON, Canada) and oligo dT_20_ following the manufacturer’s instructions. All amplifications were also performed without the addition of reverse transcriptase to confirm the absence of genomic DNA.

To obtain partial *Hsp40 type 2* cDNA from *A*. *franciscana*, the NCBI Expressed Sequence Tag (EST) database was searched for *Hsp40 type 2* sequences using a *Daphnia pulex* Hsp40-2 cDNA sequence (Accession number EFX80146.1) as reference. The sequences obtained were then BLASTED to the *A*. *franciscana* genome Online Resource for Community Annotation of Eukaryotes (ORCAE) database to identify *Hsp40 type 2* cDNAs. Primers containing the restriction enzyme sites, BamH1 and HindIII, were designed based on sequence comparisons (Integrated DNA Technologies, Coralville, IA, USA) (Table 1) and used for the amplification of full length *A*. *franciscana type 2 Hsp40* cDNA from 5 h cysts. The PCR products, resolved in 1.0% agarose gels and purified with the Wizard® SV Gel and PCR Clean-Up System (Promega, Madison, WI, USA), was then ligated into a TA vector (TOPO TA Cloning Kit, Life Technologies, Burlington, ON, Canada) and used to transform competent TOP10 *Escherichia coli* (Invitrogen, Burlington, ON, Canada). Recombinant TA vectors containing cDNA inserts of the appropriate size were isolated with the GenElute™ Plasmid Miniprep Kit (Sigma-Aldrich, Oakville, ON, Canada), and the inserts were sequenced (DNA Sequencing Facility, Centre for Applied Genomics, Hospital for Sick Children, Toronto, ON, Canada) revealing a full-length type 2 Hsp40 cDNA termed *ArHsp40-2*.

**Table 1:** Primers used for PCR.

Primers	Sequences (5'-3')	Tm (°C)
Full-length *ArHsp40-2* Forward	CGATGGGGATCCATGGGAAAGGATTTCTATAAAATAC*	54.0
Full-length *ArHsp40-2* Reverse	GCCGGATCAAGCTTTCAATTAGGCAAGGCATCACG*	54.0
qPCR *ArHsp40-2* Forward	TGACCCATTCGGTGGGTTTG	54.0
qPCR *ArHsp40-2* Reverse	TCGTGTTCAATGGGTGGGTC	54.0
qPCR α-tubulin Forward^a^	CGACCATAAAAGCGCAGTCA	49.0
qPCR α-tubulin Reverse^a^	CTACCCAGCACCACAGGTCTCT	49.0

Primers were produced by Integrated DNA Technologies (IDT), Coralville, IA, USA.

* Underlined sequences indicate restriction enzyme sites

^a^ King et al. 2013

### Sequence and structural analysis of ArHsp40-2

Nucleotide and deduced amino acid sequences, the latter obtained with ExPASY http://www.expasy.org/spdbv, were submitted to the National Center of Biotechnology International (NCBI) database for BLASTN and BLASTP searches http://www-ncbi-nlm-nih-gov.ezproxy.library.dal.ca/. Multiple sequence alignments of ArHsp40-2 against several other eukaryotic type 2 J-proteins were made with Clustal Omega version 2.0 **https://www.ebi.ac.uk/Tools/msa/clustalo/**. Protein structure models were generated using PyMol software version 2.1 https://pymol.org/2/ and functional domain analysis were computed using InterPro version 67.0 https://www.ebi.ac.uk/interpro/.

### Preparation of antibody to ArHsp40-2

A polyclonal antibody against ArHsp40-2 peptide 277-DALCGTKVDVPTLSGE-292 (Anti40-type 2) was raised in rabbits (Abiocode, Agoura Hills, CA, USA) and tested for specificity with Hsp40s produced in *Escherichia coli* and in protein extracts from *A*. *franciscana* cysts and nauplii.

For the production of full length recombinant ArHsp40-2, cDNA for the protein was generated by PCR using Platinum *Taq* Polymerase (Invitrogen, Burlington, ON, Canada), 0.2 mM primers containing restriction enzyme sites for BamH1 and HindIII (Table 1) and 0.5 μg of cDNA from *A*. *franciscana* cysts developed for 5 h as template using the reaction: 5 min at 94°C, 30 cycles of 30 s at 94°C, 30 s at 54°C, and 1 min at 72°C, followed by 10 min at 72°C. The cDNA was digested with BamH1 and HindIII at 37°C overnight and then purified with Wizard® SV PCR Clean-Up System (Promega, Madison, WI, USA). The digested cDNA was ligated into the His-tagged prokaryotic expression vector pRSET C (Invitrogen) that had been digested with BamH1 and HindIII and the recombinant plasmids were transformed into *E*. *coli* BL21(DE3) pLysS (Invitrogen) for purification. The synthesis of ArHsp40 and ArHsp40-2 was induced with 1 mM isopropyl thio-ß-D-galactoside (IPTG) for 6 h at 37°C and the recombinant Hsp40s were purified from cell free extracts of *E*. *coli* with the MagneHis™ Protein Purification System (Promega, Madison, WI, USA). ArHsp40 was obtained in the same manner using a full-length cDNA prepared previously [29].

To prepare protein extracts from shrimp, 100 mg of 0 h *A*. *franciscana* cysts and 1^st^ instar nauplii, recovered separately from seawater and frozen as described above, were homogenized on ice in 100 μl of Pipes buffer (100 mM Pipes, 1 mM MgCl_2_, 1 mM EGTA, pH 7.4) containing protease inhibitors (Halt Protease Cocktail, #87,786, Pierce Biotechnology, Rockford, IL, USA) in a 1:100 ratio (v/v) with homogenate and centrifuged at 12,000g for 10 min at 4°C. After determination of protein concentration by the Bradford assay [31] samples of extracts from 0 h cysts and 1^st^ instar nauplii and His-tagged recombinant ArHsp40 purified as described above were diluted in 4 X treatment buffer (250 mM Tris, 280 mM SDS, 40% (v/v) glycerol, 20% (v/v) ß-mercaptoethanol, 0.2% (w/v) bromophenol blue, pH 6.8), placed in a boiling water bath for 5 min and centrifuged at 10,000g for 10 min at 4°C.

To test antibody specificity 10 μg of *A*. *franciscana* cell free protein extract, 1 μg of each purified recombinant Hsp40 and 5 μl Pink Plus Pre-stained Protein Ladder (FroggaBio Inc., Toronto, ON, Canada) were resolved in 12.5% SDS polyacrylamide gels, transferred to 0.2 μm nitrocellulose membranes (BioRad, Mississauga, ON, Canada) over night at 100 mAmps and blocked for 1 h at room temperature in 5% (w/v) Carnation low fat milk powder in TBS (10 mM Tris, 140 mM NaCl, pH 7.4). Blocked membranes were then probed for 15 min at room temperature with the antibodies to *A*. *franciscana* Hsp40s diluted 1:1,000 in TBS. Membranes were washed after incubation in primary antibody for 1, 2, 3 and 4 min in TBS-T (10 mM Tris, 140 mM NaCl, 0.1% (V/V) Tween-20, pH 7.4). Subsequent to washing, membranes were incubated for 20 min at room temperature with HRP-conjugated goat anti-rabbit IgG antibody (Sigma-Aldrich, Oakville, ON, Canada) diluted 1:10,000 in TBS and then washed for 1, 2, 4 and 4 min in TBS-T followed by a final 3 min wash in TBS. Antibody-reactive proteins were visualized with Clarity ECL^TM^ Western Blotting Substrate (BioRad, Mississauga, ON, Canada) and a MF-Chemi-BIS 3.2 gel documentation system (DNR Bio-Imaging Systems, Neve, Israel).

### Quantization of ArHsp40-2 mRNA in post-diapause *A*. *franciscana*

One mg of each of the seven different *A*. *franciscana* life history stages examined (Fig 1) was harvested and flash frozen as described above prior to homogenization in 500 μl of TRIzol® (Invitrogen) for RNA extraction. RNA was quantified as described before and 0.1 μg mRNA was used as template for synthesizing cDNA with the SuperScript® III First-Strand Synthesis Kit for RT-PCR (Invitrogen) as described above.

For qPCR, forward and reverse primers (Table 1) were used to amplify an *ArHsp40-2* cDNA fragment of 144 bp, and an *α-tubulin* cDNA fragment of 276 bp, the latter as internal standard. Primers were at 1 mM and 0.5 μl of cDNA was employed as template using the following reaction: 5 min at 94°C, 30 cycles of 30 s at 94°C, 30 s at 54°C for *ArHsp40-2* (49°C for *α-tubulin*), and 1 min at 72°C, followed by 10 min at 72°C in a Rotor-Gene RG-3000 (Corbett Research, Sydney, NSW, Australia). qPCR was conducted with a QuantiFast® SYBR® Green PCR Kit (Qiagen, Mississauga, ON, Canada). Melt curve analysis were calculated by Rotor-Gene 6 Software. qPCR experiments with an efficiency of 90% or greater were accepted for analysis (Corbett Research, Sydney, NSW, Australia).

The mRNA copy numbers for *ArHsp40-2* and *α-tubulin* were calculated using standard curves, R^2^>0.99 [9]. Standard curves were generated by PCR using 0.5 μl cDNA with Platinum PCR supermix (Qiagen) and 0.4 mM primers for *ArHsp40-2* and *α-tubulin* (Table 1). The PCR product concentrations were determined by measuring the absorbance at 260 nm, and the copy number calculated based on the length of the PCR products using a base pair mass of 650 Da (**http://cels.uri.edu/gsc/cdna.html**). The cDNA standards were diluted in a 10-fold series with TE buffer (10 mM Tris, 1 mM EDTA, pH 8.0), and 0.5 μl of each dilution was used as template with the QuantiTect® SYBR® Green PCR Kit (Qiagen). All cDNA samples were assayed in duplicate. The Ct values employed to create the standard curves were fitted by linear regression for *ArHsp40-2* and *α-tubulin* mRNAs.

### Quantization of ArHsp40-2 in post-diapause *A*. *franciscana*

One-hundred mg of *A*. *franciscana* at the 7 life history stages examined (Fig 1) was harvested and flash frozen before homogenization on ice in 100 μl of Pipes buffer and centrifugation as described above. After determination of protein concentration by the Bradford assay [31] samples were diluted in 4 X treatment buffer, placed in a boiling water bath for 5 min and centrifuged at 10,000g for 10 min at 4°C.

Forty μg of protein from each supernatant was resolved in 12.5% SDS polyacrylamide gels and either stained with Colloidal Coomassie Blue (10% (w/v), ammonium sulfate, 0.1% (w/v), Coomassie G-250, 3% (v/v), phosphoric acid, 20% (v/v), ethanol) or transferred to 0.2 μm nitrocellulose membranes (BioRad, Mississauga) overnight at 100 mAmp and blocked for 1 h at room temperature in 5% (w/v) Carnation low fat milk powder in TBS. Subsequent to blocking, membranes were probed for 15 min at room temperature with either Anti40-type 2 diluted 1:1000 in TBS or Anti-Y, a polyclonal antibody raised in rabbit to tyrosinated α-tubulin [32] diluted 1:1,000 in TBS as described above.

### Heat induction of ArHsp40-2 in *A*. *franciscana* larvae

First instar *A*. *franciscana* nauplii grown at 27°C in seawater were collected 4 h after hatching when they contained reduced amounts of ArHsp40-2, and then incubated in 20 ml seawater in Corex tubes at 39°C for 1 h in a programmable water bath (VWR International LLC, Mississauga, ON, Canada) [1]. Heat shocked animals were harvested on nylon mesh filters, frozen and either homogenized on ice with protease inhibitors in Pipes buffer as described before or incubated at 27°C for 2, 4, 6, and 8 h for recovery and then collected and homogenized. Forty μg of each cell-free protein homogenate was resolved in SDS polyacrylamide gels, transferred to nitrocellulose, blocked and probed with either Anti40-type2 or Anti-Y prior to washing and imaging as described above.

### Image analysis

Images of developing animals were captured using a Nikon AZ100 microscope. Images and figures were prepared for publication using Photoshop (Adobe Creative Cloud; Adobe Systems Inc). Bands of immunoreactive proteins on western blots were quantified with Image Studio Software (Li-Core Biosciences, Lincoln, NE, USA), and band intensities for ArHsp40-2 were compared to band intensities for tyrosinated α-tubulin at each development stage examined.

### Statistical analysis

One-way ANOVA followed by a Dunnett’s test was carried out in order to detect significant difference between all means from control means. All data were plotted as means +/- SE unless otherwise stated. Analyses were carried out using GraphPad Prism 5 software (GraphPad Software Inc., La Jolla, CA, USA).

## Results

### *ArHsp40-2* cDNA

**Fig 2:**
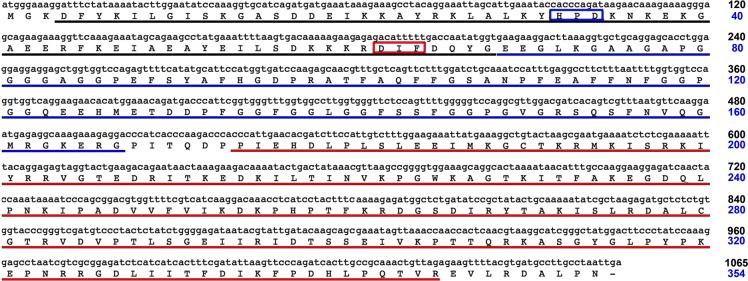
ArHsp40-2 sequence. Nucleotide and amino acid sequences for the full length open reading frame of ArHsp40-2. J-domain, underlined in black; HPD motif, boxed in blue; G/F rich region, underlined in blue; DIF motif, boxed in red; C-terminal domain, underlined in red. The number of nucleotides and amino acid residues is indicated on the right.

**Fig 3:**
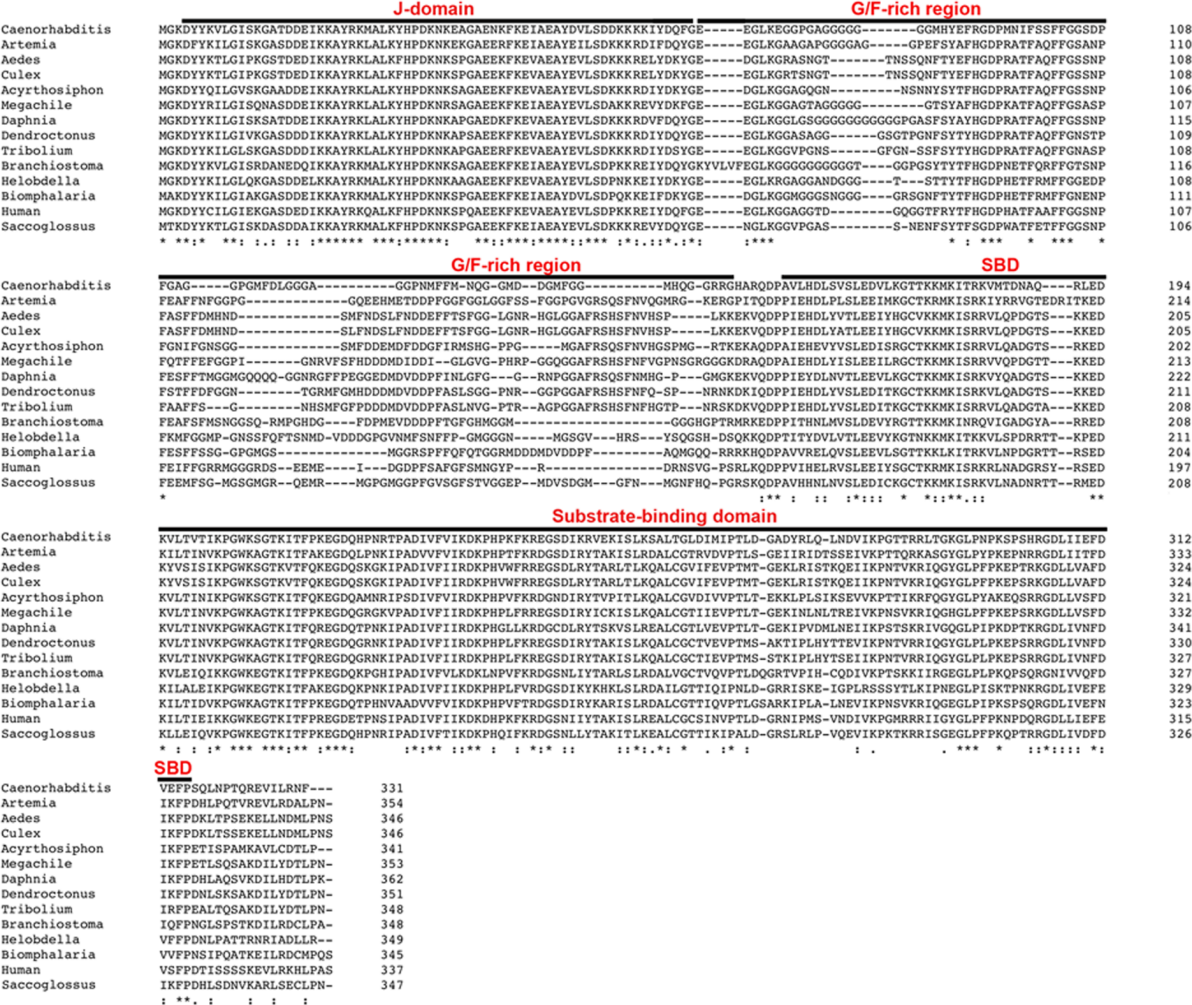
Sequence alignment of ArHsp40-2 with type 2 Hsp40s from other organisms. The amino acid sequence of ArHsp40-2 from *A*. *franciscana* was aligned with type 2 Hsp40s from the organisms listed in Table 2. The domains of ArHsp40-2 are over-lined in black and labeled in red. SBD, substrate binding domain. Identical amino acid residues are indicated by an asterisk, similar residues by a colon and semi-conserved residues by a period. The number of amino acid residues in each sequence is indicated on the right.

**Fig 4:**
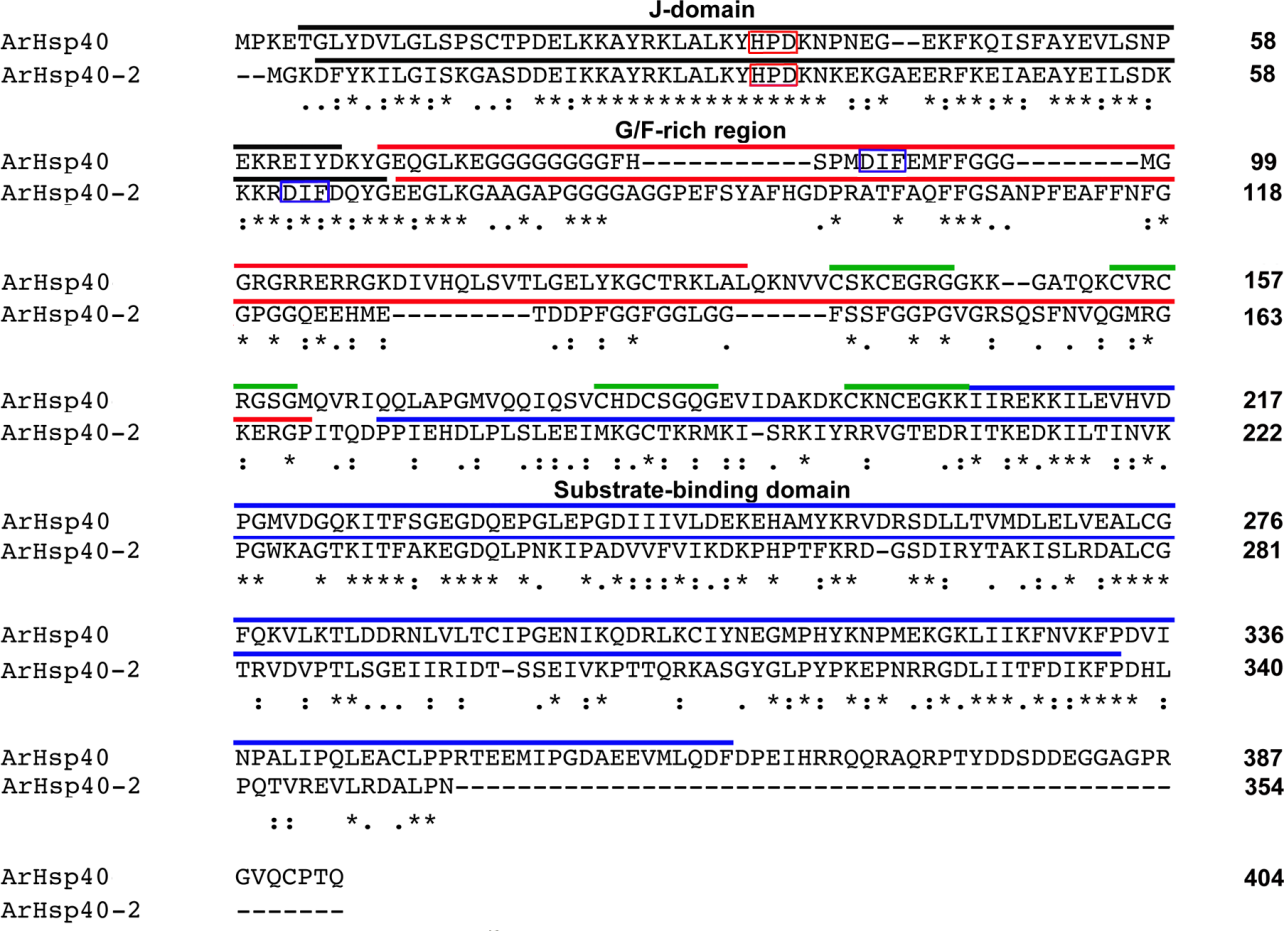
Sequence alignment of ArHsp40 and ArHsp40-2. The amino acid sequences of ArHsp40 and ArHsp40-2 were compared by CLUSTAL OMEGA (**http://www.ebi.ac.uk/clustalw2**). J-domain, over-line black; HPD motifs (red boxes); G/F rich region, over-line red; DIF motifs, blue boxes; CXXCXGXG motifs of the zinc binding domain in ArHsp40 only, green over-line; C-terminal domain, blue over-line. Identical amino acid residues are indicated by an asterisk, similar residues by a colon, semi-conserved residues by a period, and no residue by a dash. The number of amino acid residues in each sequence is indicated on the right.

**Fig 5:**
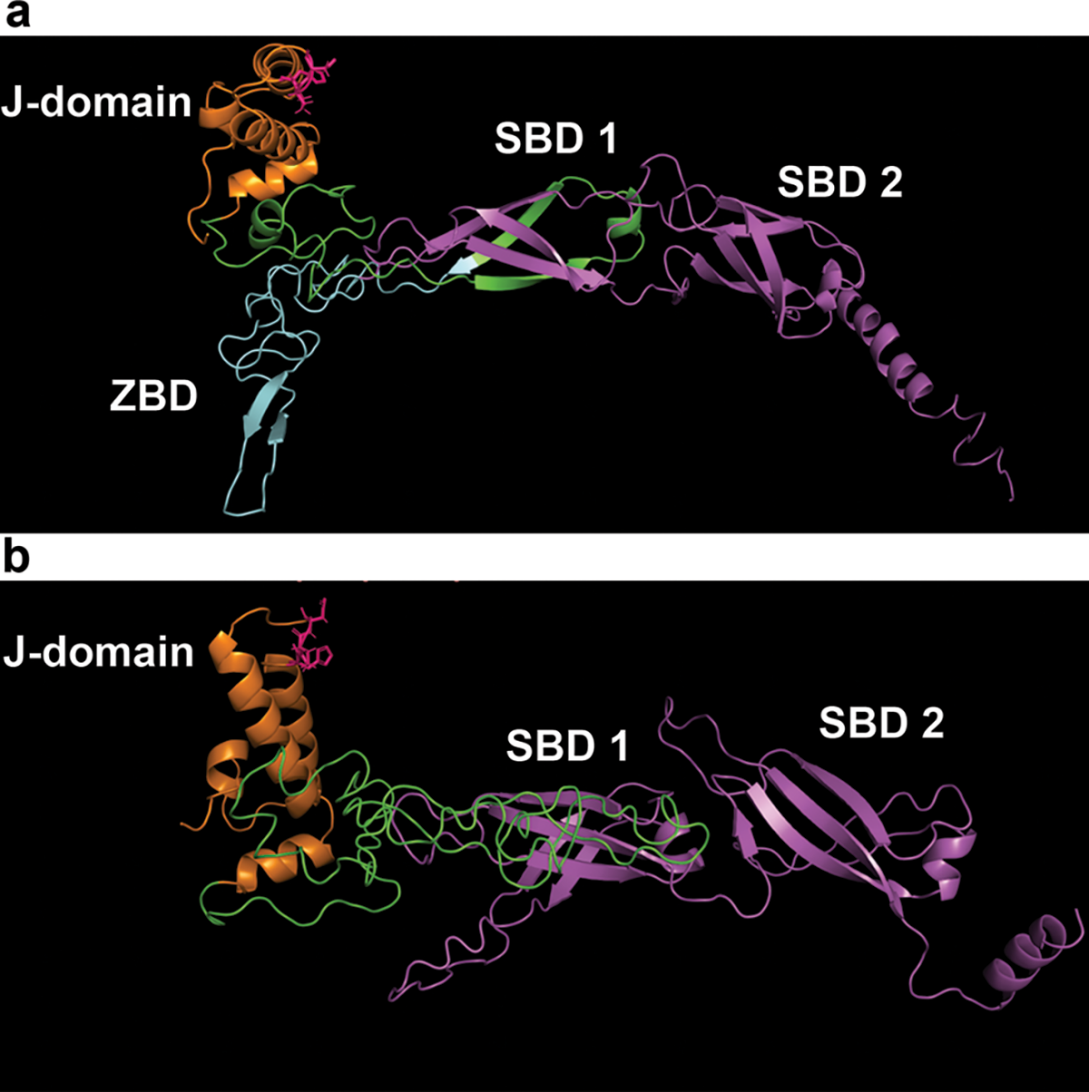
Protein modeling of ArHsp40 and ArHsp40-2. The structures of ArHsp40 (a) and ArHsp40-2 (b), displayed with the amino-terminus to the left, were generated by PyMol https://pymol.org/2/ software. J-domain, orange; HPD motif, pink; G/F rich region, green; zinc binding domain (ZBD), cyan (ArHsp40 only); carboxyl-terminal substrate binding domain, magenta, contains double β-barrel structures termed substrate binding domains 1 and 2 (SBD1, SBD2), followed by the dimerization domain.

*ArHsp40-2* cDNA (Accession number MH138022) from 5 h cysts cloned by RT-PCR consisted of 1065 bp which encoded a 354-amino acid polypeptide with a predicted molecular mass of 38.9 kDa and a theoretical pI of 8.99 (Fig 2). ArHsp40-2 possessed a conserved N-terminal J-domain with a HPD motif, followed by a Gly/Phe (G/F) rich region with a conserved DIF motif and then a carboxyl-terminal domain. ArHsp40-2 lacked a zinc binding domain containing CXXCXGXG motifs and it shared significant sequence similarity, except in the G/F rich region, with type 2 J-domain proteins from several animals (Fig 3 and Table 2). ArHsp40 [29] and ArHsp40-2 possessed 64% similarity and 36% identity (Fig 4) with ArHsp40-2 having a deletion of 53 amino acid residues at its carboxyl terminus as compared to ArHsp40. The carboxyl-terminal domain contained a double barrel β-domain followed by an α-helix which forms a dimerization domain similar to that found in ArHsp40 and other J-domain proteins (Fig 5).

**Table 2:** Comparison of ArHsp40-2 with type 2 Hsp40s from other organisms.

Species	Class	Identitiy (%)*	Accession Number
*Daphnia pulex*	Branchiopoda	64	EFX80146.1
*Caenorhabditis elegans*	Chromadorea	54	NP_496468.1
*Helobdella robusta*	Citellata	52	XP_009022917.1
*Saccoglossus kowalevskii*	Enteropneusta	58	XP_002732367.1
*Biomphalaria gladrata*	Gastropoda	55	XP_013081596.1
*Aedes aegypti*	Insecta	58	XP_001658074.1
*Culex quinquefasclatus*	Insecta	58	XP_001845463.1
*Acyrthosiphon pisum*	Insecta	60	XP_001949061.2
*Megachile rotundata*	Insecta	62	XP_003707298.1
*Dendroctonus ponderosae*	Insecta	65	XP_019765112.1
*Tribolium castaneum*	Insecta	63	XP_008200761.1
*Branchiostoma floridae*	Leptocardii	52	XP_002601425.1
*Homo sapiens*	Mammalia	58	NP_008965.2

*The % identity between ArHsp40-Type 2 and type 2 Hsp40s from different organisms was determined by BLASTP at **http://www.ncbi.nlm.nih.gov**.

### Anti40-type1 and Anti40-type2 immunoreactivities

**Fig 6:**
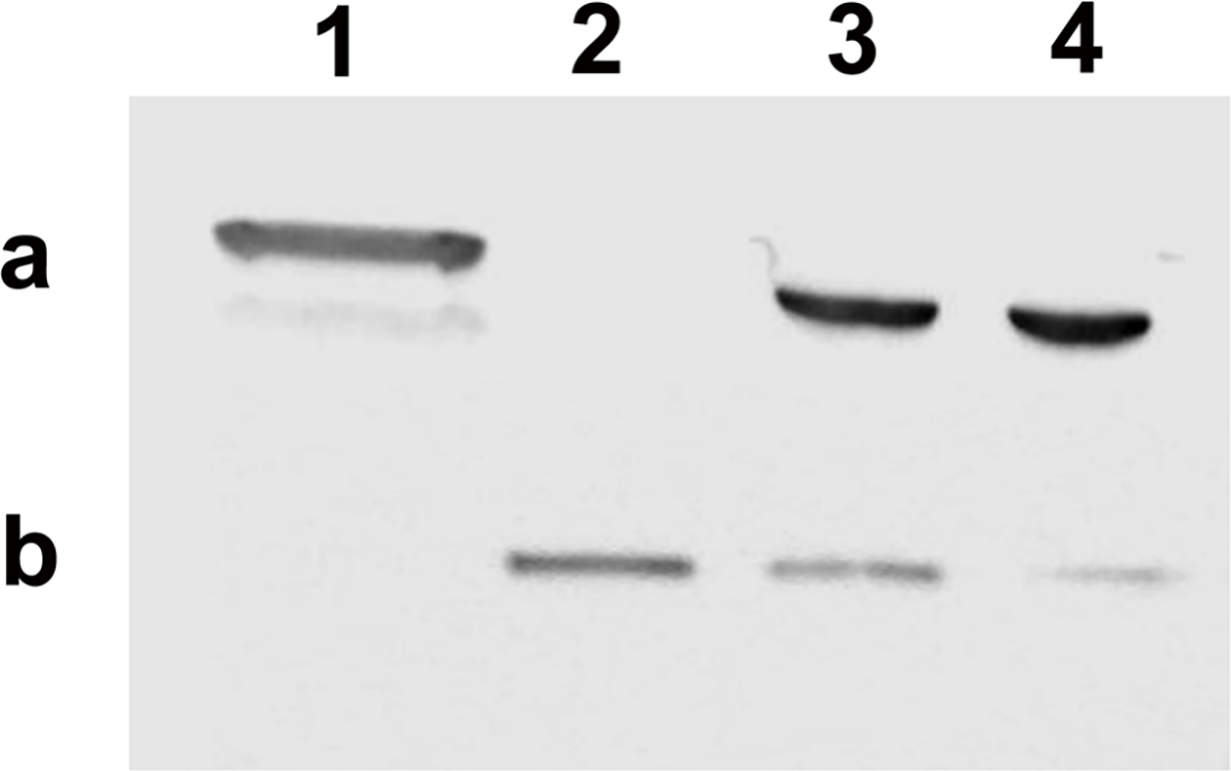
Specificity of antibodies raised to ArHsp40 and ArHsp40-2. Protein extracts from *A*. *franciscana* and recombinant ArHsp40s were resolved in SDS polyacrylamide gels, blotted to nitrocellulose and stained with antibodies. Lane 1, 1 μg of recombinant ArHsp40; 2, 1 μg of recombinant ArHsp40-2; 3, 20 μg of protein extract from *A*. *franciscana* cysts; 4, 20 μg of protein extract from *A*. *franciscana* instar 1 nauplii. Blots were probed with Anti40-type 1 (a) and Anti40-type 2 (b).

Anti40-type 1 [29] reacted with purified recombinant ArHsp40 but not with purified recombinant ArHsp40-2 (Fig 6). Conversely, Anti40-type2 reacted with purified recombinant ArHsp40-2 but not with purified recombinant ArHsp40 (Fig 6). Both antibodies recognized co-migrating polypeptides of approximately 38.9 kDa in protein extracts from *A*. *franciscana* cysts and first instar nauplii S1 Fig.

### The post-diapause synthesis of *ArHsp40-2* was developmentally regulated

**Fig 7:**
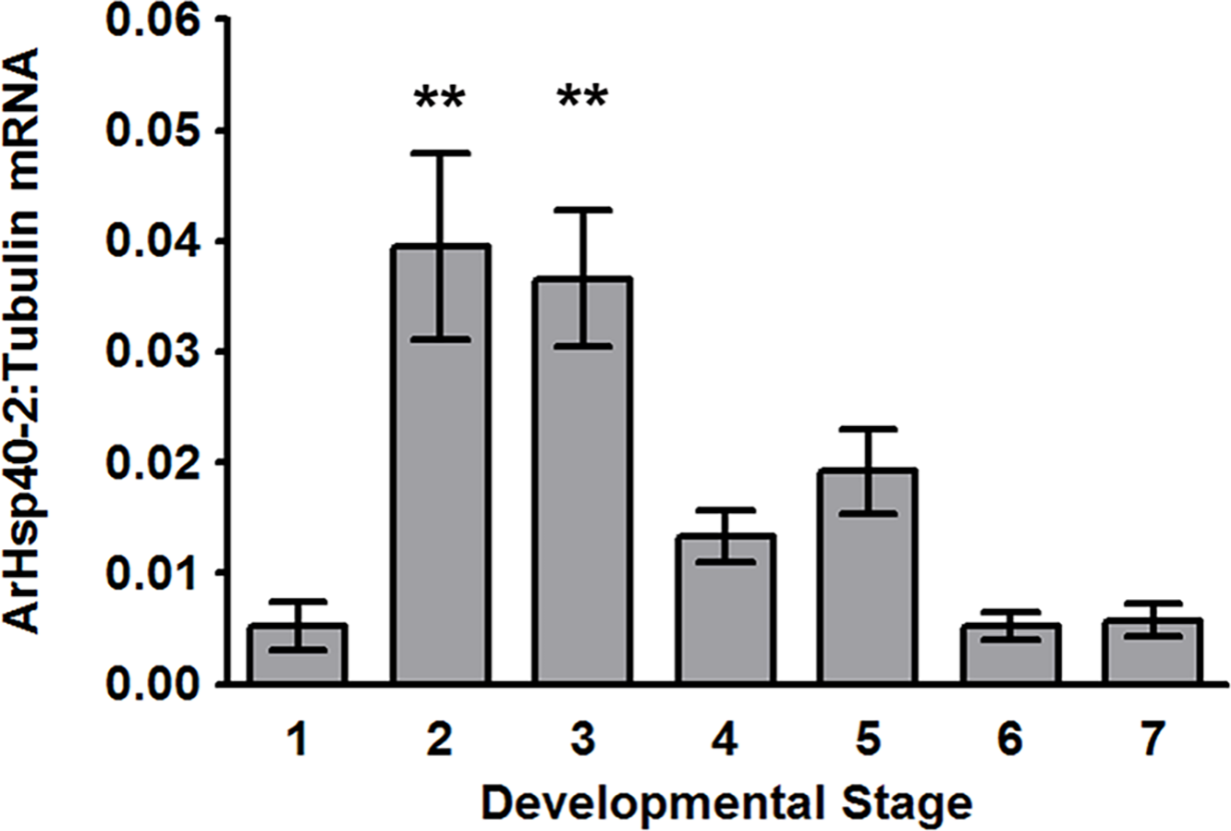
ArHsp40-2 mRNA was developmentally regulated during post-diapause development of *A*. *franciscana*. The amount of *ArHsp40-2* mRNA determined by qPCR was normalized to *α-tubulin* mRNA in post-diapause *A*. *franciscana*. Bar 1, 0 h cysts; 2, 5 h cysts; 3, 10 h cysts/E1; 4, E2/E3; 5, instar 1 nauplii; 6, early instar 2 nauplii; 7, late instar 2 nauplii. The experiment was performed in duplicate with RNA samples from 3 replicates of each life history stage. The amount of *ArHsp40-2* mRNA in life history stages indicated by bars labeled with asterisks are significantly different from the amount of *ArHsp40-2* mRNA in 0 h cysts, **, *P*<0.01. Error bars represent standard error of 3 replicates per experiment.

**Fig 8:**
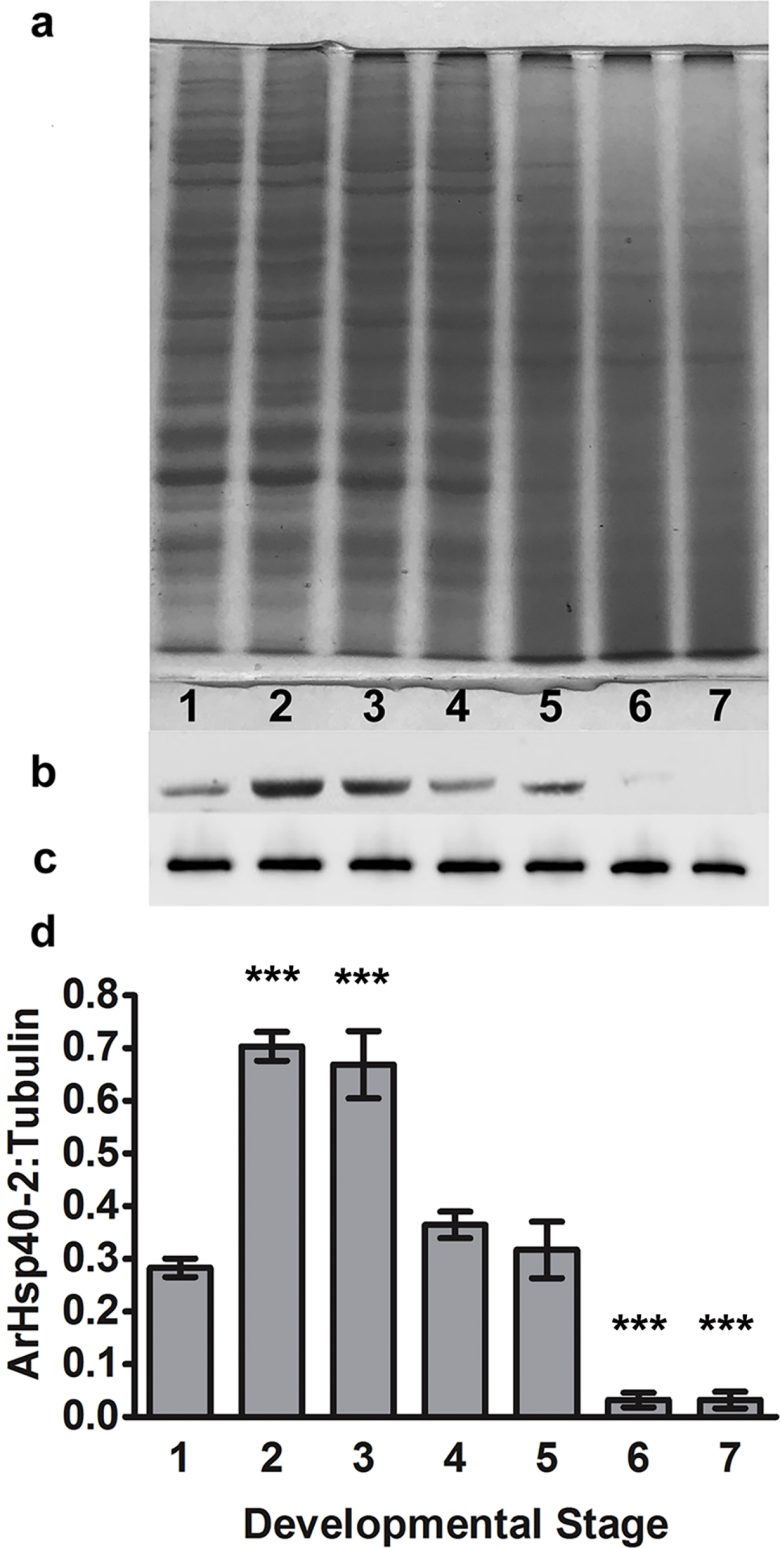
ArHsp40-2 was developmentally regulated during post-diapause development of *A*. *franciscana*. Cell-free homogenates from post-diapause *A*. *franciscana* were resolved in SDS-polyacrylamide gels and either stained with Colloidal Coomassie blue (a) or blotted to nitrocellulose and probed with either Anti40-type2 (b) or anti-Y (c). The antibody-reactive protein bands were quantified with Image Studio Software and the ratio of ArHsp40-2:tyrosinated α-tubulin was determined. Lane 1, 0 h cysts; 2, 5 h cysts; 3, 10 h cysts/E1; 4, E2/E3; 5, instar 1 nauplii; 6, early instar 2 nauplii; 7, late instar 2 nauplii. The amount of ArHsp40-2 in life history stages indicated by bars labeled with asterisks are significantly different from the amount of ArHsp40-2 in 0 h cysts, ***, *P*<0.005. Error bars represent standard error of 3 replicates per experiment.

qPCR and immunoprobing of western blots using α-tubulin mRNA and tyrosinated α-tubulin respectively as internal controls showed that the amounts of *ArHsp40-2* mRNA (Fig 7) and protein (Fig 8), as compared to 0 h cysts, increased significantly in 5 h cysts and 10 h cysts/E1. Subsequently, *ArHsp40-2* mRNA and ArHsp40 decreased in E2/E3 nauplii, declining to levels similar to or below that in 0 h cysts as development progressed. There were no significant changes in tyrosinated α-tubulin S2 Fig.

### ArHsp40-2 synthesis was induced by heat shock in *A*. *franciscana* larvae

**Fig 9:**
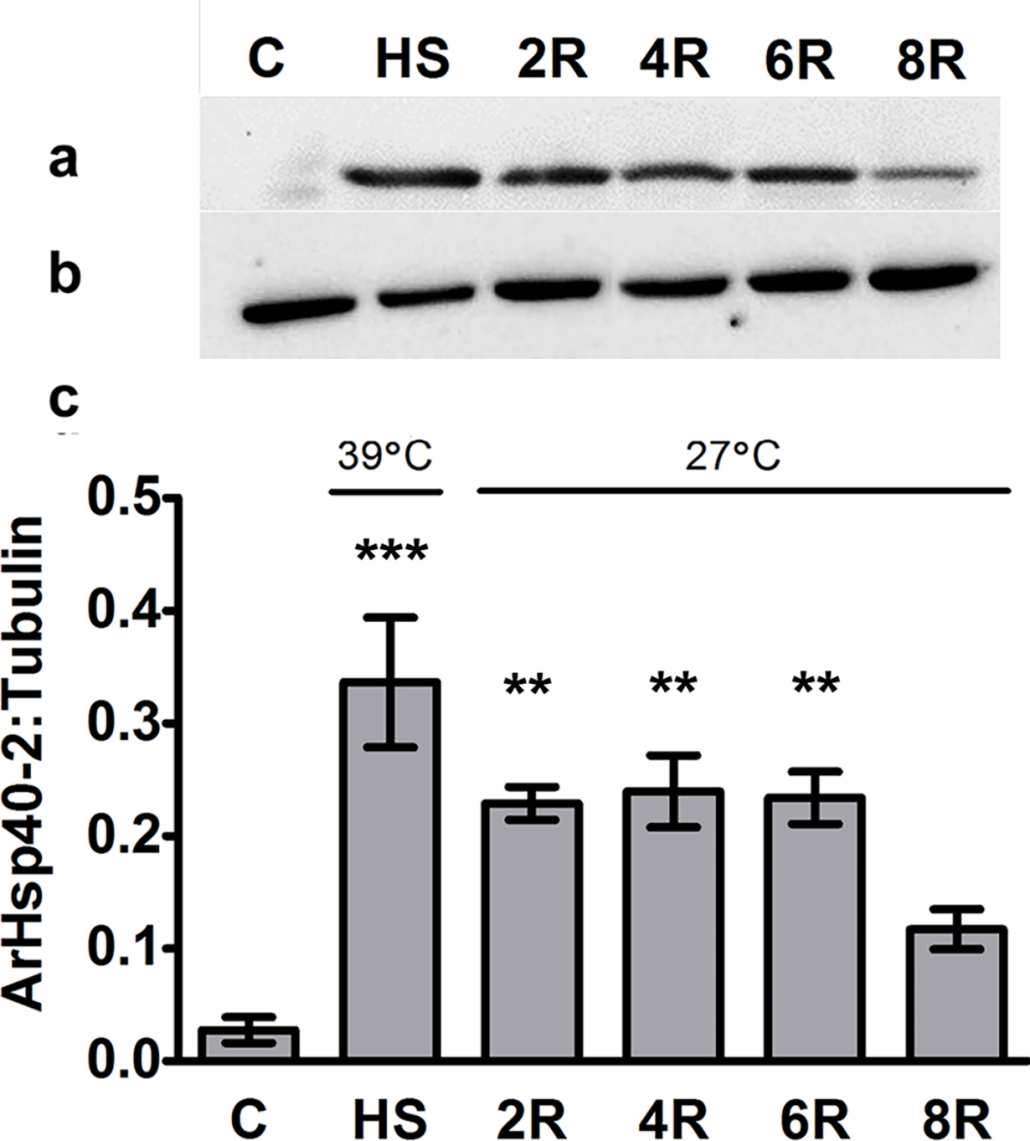
ArHsp40-2 was induced by heat shock in post-diapause *A*. *franciscana* nauplii. Cell-free homogenates from heat shocked instar 1 nauplii were resolved in SDS-polyacrylamide gels, blotted to nitrocellulose and probed with either Anti40-type2 (a) or anti-Y (b). The antibody-reactive protein bands were quantified with Image Studio Software and the ratio of ArHsp40-2:tyrosinated α-tubulin was calculated (c). Lane C, no heat shock; HS, 1 h at 39°C; 2R, 4R, 6R, 8R, recovery at 27°C for 2, 4, 6 and 8 h respectively. The amount of ArHsp40-2 in life history stages indicated by bars labeled with asterisks are significantly different from the amount of ArHsp40-2 in 0 h cysts, **, *P*<0.01; ***, *P*<0.005. Error bars represent standard error of 3 replicates per experiment.

ArHsp40-2 increased almost 10-fold in 1^st^ instar nauplii heat shocked at 39°C for 1 h and remained elevated for 6 h of recovery before declining (Fig 9) S3 Fig.

## Discussion

Diapausing cysts of *A*. *franciscana* survive long periods of time in an essentially ametabolic state where they are resistant to stresses such as heat, cold, anoxia, oxidation and desiccation [5, 33–37]. In preparation for diapause, embryos of *A*. *franciscana* up-regulate synthesis of the sHsp p26 [1, 8], which is thought to sequester denaturing proteins when ATP concentration is low and ATP-dependent molecular chaperones are unable to fold proteins. RNAi experiments confirm that p26 has an important role in protecting diapause cysts during stress [9], but because this sHsp lacks foldase activity other molecular chaperones including Hsp110, Hsp90 and Hsp70, and their accessory proteins such as the Hsp40s, are required to refold proteins sequestered by p26. Hsp70 and Hsp90 are synthesized in *A*. *franciscana* and *A*. *sinica* embryos that are destined to enter diapause and they are available to maintain protein homoeostasis during embryogenesis and post-diapause development [6, 35, 38–41]. Additionally, embryos fated to become nauplii up-regulate mRNA for Hsp60, Hsp70, Hsp90 and Hsp110 [39] and Hsp70 increases in nauplii under heat stress where it can play a role in resisting infection by *Vibrio campbellii* [42, 43].

Little is known about Hsp40s in *A*. *franciscana* and other crustaceans undergoing diapause, but they are up-regulated in insects during diapause. For example, in early diapause of the flesh fly, *Sarcophaga crassipalpis* Hsp40 in the brain is up-regulated and hyper-phosphorylated [44, 45], but how this contributes to diapause in uncertain. *Hsp40* mRNA increases upon diapause entry and metabolic depression in the silk worm *Bombyx mori*, as is true for *Hsp70* and *Hsc70* mRNAs [46]. The synthesis of *Hsp40* mRNA is also stimulated in insects by stress. As a case in point, Hsp40 mRNA is induced by heat in the leaf miner *Liriomyza sativa* and by cold in *Drosophila melanogaster* [47–49]. Larvae of the gall fly *Eurosta solidaginis* synthesize Hsp40 when exposed to freezing [50] and *Hsp40* mRNA is up-regulated in *S*. *crassipalpis* and *D*. *melanogaster* adults under oxidative stress [51, 52].

We characterized an *A*. *franciscana* J-domain protein named ArHsp40 and showed that it is likely to play a role in stress tolerance during diapause and to influence post-diapause development [29]. To expand the study of Hsp40s in *A*. *franciscana*, a second J-domain protein termed ArHsp40-2 has been identified and it is described herein. Unlike ArHsp40, ArHsp40-2 lacks a zinc binding domain, although the two proteins share significant sequence similarity and structural elements, demonstrating that they are related. Multi-sequence alignment showed that ArHsp40-2 and type 2 Hsp40s from divergent species are similar, other than in the GF-rich region.

*ArHsp40-2* mRNA, present in relatively low amounts in 0 h cysts, increased approximately eight-fold during the first five hours of post-diapause development and remained elevated until cyst shells cracked and membrane-enclosed nauplii emerged. The augmentation in mRNA indicates transcriptional regulation of the *ArHsp40-2* gene during early post-diapause development in *A*. *franciscana*, although other possibilities such as an increase in *ArHsp40-2* mRNA stability cannot be ruled out at this time. The amount of *ArHsp40-2* mRNA underwent a significant drop in E2/E3 nauplii and declined even further in instar II nauplii, implying that ArHsp40-2, if it followed the same trend as the mRNA, functions mainly during early post-diapause development, a time when proteins sequestered by p26 are required by the developing embryo. To determine the level of ArHsp40-2 during post-diapause development of *A*. *franciscana*, an antibody shown to react with purified recombinant ArHsp40-2 and a single protein in cell free extracts of cysts and nauplii, but not with purified recombinant ArHsp40, was used. The early post-diapause increase in ArHsp40-2 relative to that in 0 hour cysts was less than that seen for its mRNA but the pattern of accumulation was very similar, again indicative of ArHsp40-2 function during early post-diapause development when p26 is releasing sequestered proteins for refolding. Additionally, ArHsp40-2, like ArHsp40, was induced by heat where it had the potential to contribute to stress tolerance by transferring proteins to Hsp70 which then remain bound because ATP concentration is low.

## Conclusions

A cDNA, termed *ArHsp40-2* was cloned from *A*. *franciscana* and shown to encode a type 2 J-domain protein which lacked a zinc binding domain. ArHsp40 and ArHsp40-2 shared sequence and structural similarities, and the synthesis of ArHsp40-2 was, like ArHsp40, developmentally regulated, but their patterns of synthesis were distinct indicating dissimilar functions. Both proteins were induced by heat in nauplii. The results suggest a role for ArHsp40-2 in diapausing cysts of *A*. *franciscana* and during early post-diapause development where it assists in the rescue of proteins sequestered on p26, an abundant sHsp specific to diapause.

## Supporting information

S1 Fig Antibody specificity. Left panel, test of Anti40-1, panel a in the manuscript figure; right panel, test of Anti40-2, the middle 4 lanes of the blot shown in the right panel were used to generate panel B in the manuscript figure–unused lanes in the right panel were duplicates.
(TIF)

S2 Fig ArHsp40-2 developmental regulation. Left panel, Commassie-stained SDS-polyacrylamide gel; middle panel, ArHsp40-2, blot was truncated at the time of staining, the positions of lanes 5 and 6 were reversed for the manuscript figure as the samples were loaded in the wrong order; right panel, tubulin, the first two lanes on the left were not used in the manuscript figure.
(TIF)

S3 Fig ArHsp40-2 heat shock response. Left panel, Commassie-stained SDS polyacrylamide gel, not used in manuscript figure; middle panel, ArHsp40-2, blot was trunccated at the time of staining; right panel, tubulin, centre lane removed as the well did not load properly.
(TIF)
