# Supplementary figures and images for: Post-diapause synthesis of ArHsp40-2, a type 2 J-domain protein from Artemia franciscana, is developmentally regulated and induced by stress
Source: PLoS One. 2018 Jul 26;13(7):e0201477. doi: 10.1371/journal.pone.0201477 (PMC6062144; doi:10.1371/journal.pone.0201477)

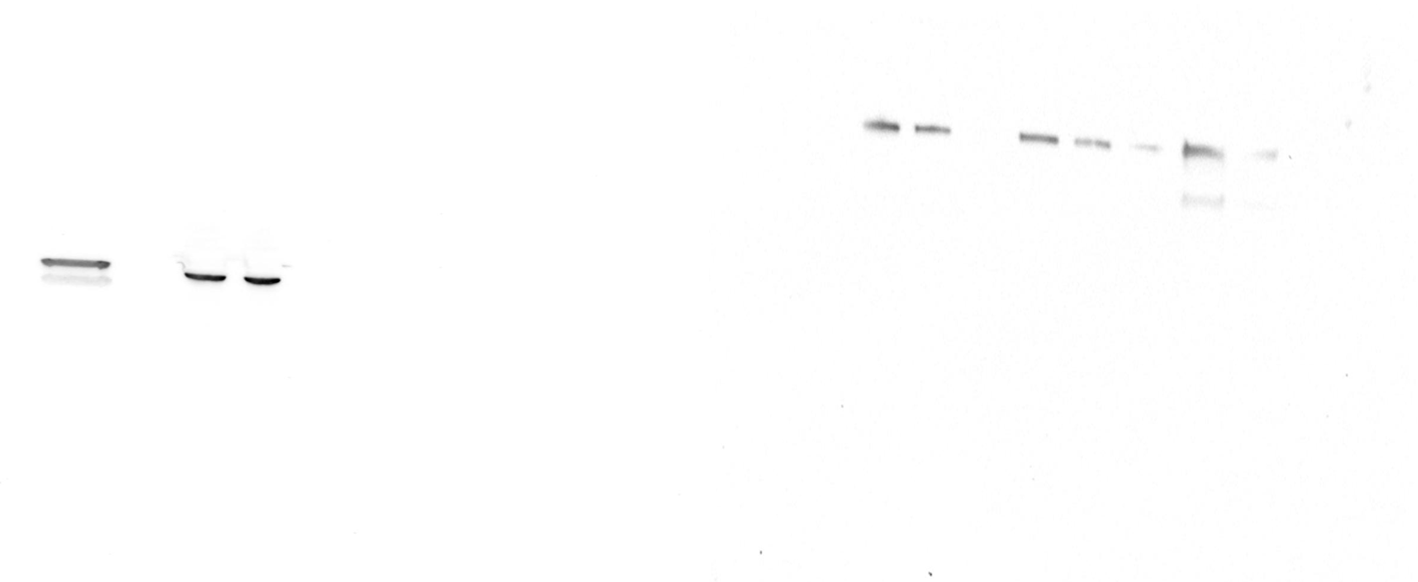

Supplement: S1 Fig — Left panel, test of Anti40-1, panel a in the manuscript figure; right panel, test of Anti40-2, the middle 4 lanes of the blot shown in the right panel were used to generate panel B in the manuscript figure–unused lanes in the right panel were duplicates. (TIF) [file pone.0201477.s001.tif]

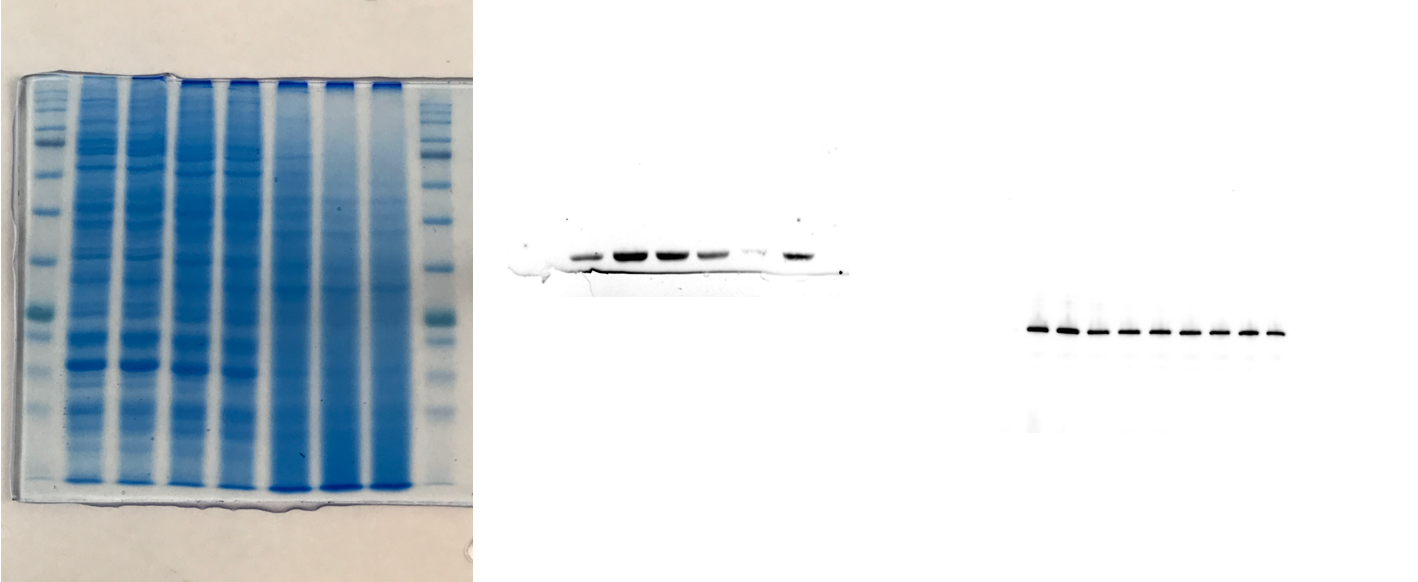

Supplement: S2 Fig — Left panel, Commassie-stained SDS-polyacrylamide gel; middle panel, ArHsp40-2, blot was truncated at the time of staining, the positions of lanes 5 and 6 were reversed for the manuscript figure as the samples were loaded in the wrong order; right panel, tubulin, the first two lanes on the left were not used in the manuscript figure. (TIF) [file pone.0201477.s002.tif]

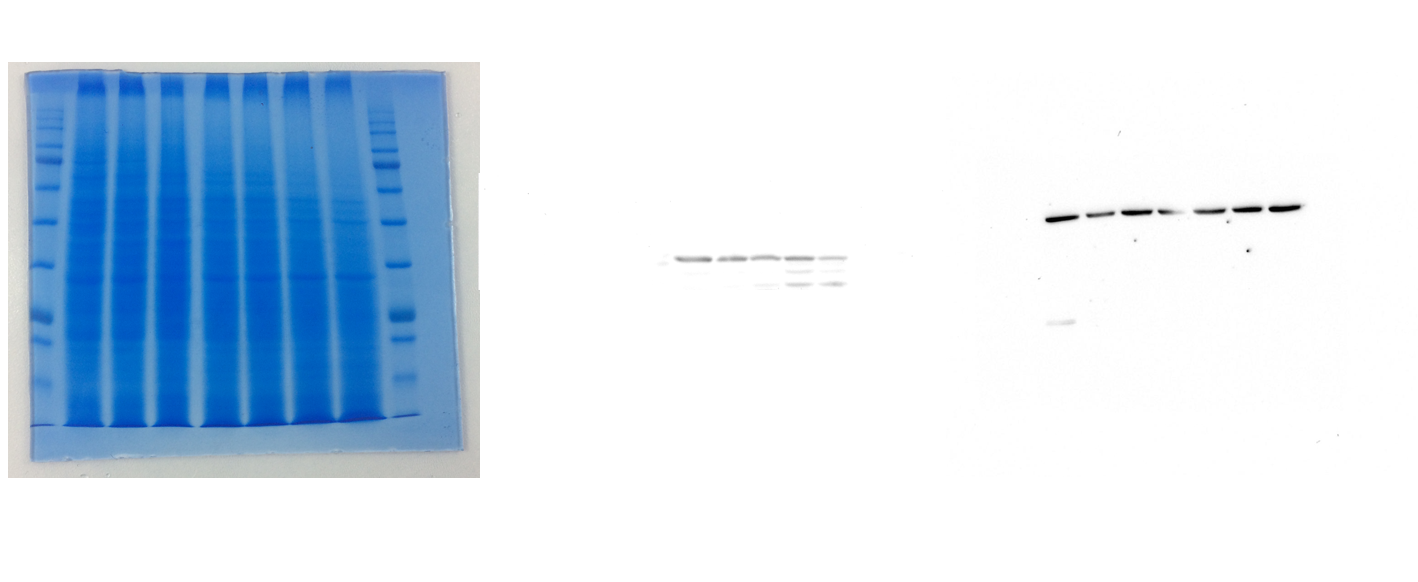

Supplement: S3 Fig — Left panel, Commassie-stained SDS polyacrylamide gel, not used in manuscript figure; middle panel, ArHsp40-2, blot was trunccated at the time of staining; right panel, tubulin, centre lane removed as the well did not load properly. (TIF) [file pone.0201477.s003.tif]
